# 25-Hydroxycholesterol Inhibits Adipogenic Differentiation of C3H10T1/2 Pluripotent Stromal Cells

**DOI:** 10.3390/ijms21020412

**Published:** 2020-01-09

**Authors:** Dorothy Moseti, Alemu Regassa, Chongxiao Chen, Karmin O, Woo Kyun Kim

**Affiliations:** 1 Department of Animal Science, University of Manitoba, 201 Animal Science building, Winnipeg, MB R3T 2N2, Canadaalemuhunde2017@gmail.com (A.R.);; 2 Department of Poultry Science, University of Georgia, 303 Poultry Science building, Athens, GA 30602-2772, USA; cxchen@uga.edu

**Keywords:** oxysterols, C3H10T1/2 stromal cells, differentiation, hedgehog signalling, PPARγ, ADD1/SREBF1

## Abstract

Understanding of adipogenesis is important to find remedies for obesity and related disorders. In addition, it is also critical in bone disorders because there is a reciprocal relationship between adipogenesis and osteogenesis in bone micro-environment. Oxysterols are pro-osteogenic and anti-adipogenic molecules via hedgehog activation in pluripotent bone marrow stomal cells. However, no study has evaluated the role of specific oxysterols in C3H10T1/2 cells, which are a good cell model for studying osteogenesis and adipogenesis in bone-marrows. Thus, we investigated the effects of specific oxysterols on adipogenesis and expression of adipogenic transcripts in C3H10T1/2 cells. Treatment of cells with DMITro significantly induced mRNA expression of Pparγ. This induction was significantly inhibited by 25-HC. The expression of *C/cepα*, *Fabp4* and *Lpl* was also inhibited by 25-HC. To determine the mechanism by which 25-HC inhibits adipogenesis, the effects of the hedgehog signalling pathway inhibitor, cyclopamine and CUR61414, were evaluated. Treatment of C3H10T1/2 cells with DMITro + cyclopamine or DMITro + CUR61414 for 96h did not modulate adipocyte differentiation; cyclopamine and CUR61414 did not reverse the inhibitory effects of 25-HC, suggesting that the canonical hedgehog signalling may not play a role in the anti-adipogenic effects of 25-HC in C3H10T1/2 cells. In addition, LXR agonist did not inhibit adipogenesis, but 25-HC strongly inhibits adipogenesis of C3H10T1/2 cells. Our observations showed that 25-HC was the most potent oxysterol in inhibiting adipogenesis and the expression of key adipogenic transcripts in C3H10T1/2 cells among the tested oxysterols, suggesting its potential application in providing an intervention in osteoporosis and obesity. We also report that the inhibitory effects of 25-HC on adipogenic differentiation in C3H10T1/2 cells are not mediated by hedgehog signaling and LXR.

## 1. Introduction

Obesity is a major health problem that leads to increased risk of type II diabetes mellitus (T2DM), cardiovascular diseases and hypertension and has been associated with high morbidity and mortality rates, especially in the industrialized world [1]. Obesity is associated with adipose tissue and the development of fat cells or adipocytes [2,3]. A study of the mechanisms of adipose tissue development and the transcription markers that influence maturation of adipocytes is important, not only to understand the pathogenesis of obesity but also to identify pathways and proteins that can be targeted for pharmacological interventions in order to combat the growing incidence of obesity [4,5]. Adipocytes play an important role in maintaining energy balance in the body of animals [6]. Excess energy is stored in form of lipids in adipocytes within the white adipose tissue, and when energy intake is scarce, this tissue is broken down and released into the blood stream in form of free fatty acids and used as a source of energy by other tissues [6,7]. However, excess energy intake leads to an increase in the adipose tissue due to an increase in the number or size of adipocytes, which then leads to obesity [8]. Excess body fats also lead to accumulation of cholesterol in arterial wall leading to atherosclerosis, a major cardiovascular disease. Furthermore, the enlarged fat cells secrete certain adipokines, which hinder insulin signalling leading to development of T2DM [7]. 

Adipogenesis involves the formation of fat cells from undifferentiated precursor cells, a process involving a transcriptional network with various transcriptional markers that coordinate the expression of a number of proteins involved in mature fat cell formation [9]. The main classes of markers that directly influence fat cell formation are peroxisome proliferator activated receptor gamma (*Pparγ*) and CCAAT/enhancer binding proteins (*C/epbs*) [10]. *Pparγ* is a member of the nuclear hormone receptor super family of ligand-activated transcription factors, plays a central role in the regulation of gene expression of various physiological processes, and is the dominant or “master” regulator of adipocyte biology [10,11,12,13]. *Pparγ* is highly expressed in adipose tissues, and its expression is significantly induced during the differentiation of preadipocytes into adipocytes. Without *Pparγ*, precursor cells are unable to differentiate and express the aspects of mature fat cells [9,14]. *Pparγ* is thus necessary for adipose tissue accumulation and function and is also a target of antidiabetic thiazolidinedione (TZD) drugs, which promote insulin sensitivity [4].

The *C/ebps* are a family of highly conserved basic-leucine zipper proteins consisting of six members of which three family members (*C/ebp α, β*, and ∂) play important roles in adipogenesis. Expression of *C/ebpβ* and *C/ebp∂* (Early markers) takes places immediately after induction of differentiation [15,16]. These transcription factors then induce the expression of *Cebpα* and *Pparγ*, which are responsible for the entire adipocyte differentiation process involving formation of lipid droplets and expression of various metabolic programs associated with mature fat cells [3]. *Cebpα* is expressed during later stages of adipogenesis, and mouse models have been used to demonstrate the importance of this transcription factor in fat cell differentiation. In these models, deletion of *Cebpα* leads to an abnormal or lack of lipid accumulation within adipocytes [16]. 

In addition, understanding of adipogenesis in bone micro-environment is also important for prevention of bone-related disorders such as osteoporosis because multipotent mesenchymal stromal cells (MSCs) in bone compartments can be differentiated into adipocytes as well as osteoblasts [17]. MSCs are another reliable tool for studying differentiation of cells into adipocytes. These cells can be isolated from animal and human tissues, grown in culture and induced to differentiate into bone, cartilage, muscle or fat cells [18]. The mouse pluripotent cell line, C3H10T1/2, which was established from 14- to 17-day-old C3H mouse embryos, displays characteristics of MSC and is a good model for studying osteogeneis and adipogenesis in bone marrows. Previous studies have shown that treatment of C3H10T1/2 cells with 5-azacytidine leads to differentiation into cells that display features of bone, skeletal and adipose tissues [19,20]. These cells display a fibroblastic morphology in culture when sub-confluent and flat epithelial like structures when fully confluent [21]. There is a reciprocal relationship between osteogenic and adipogenic differentiation in MSCs, which make MSC a more valuable and sustainable research model to investigate the pathways related to bone and fat development [22]. A potential strategy to regulate the differentiation of MSCs involves the use of oxysterols [23,24], which are products of cholesterol oxidation obtained through enzymatic and non-enzymatic processes and are found in various human tissues and fluids [25,26,27]. 

Oxysterols consist of 27 carbon atoms and are involved in many biological processes such as cholesterol homeostasis [25,27]. Oxysterols are believed to be involved in regulation of gene expression associated with lipid metabolism and play important roles in differentiation, developmental and inflammatory responses [28]. Oxysterols are found in circulation or various tissues at very low levels as cholesterol metabolites [25,26,27]. Specific oxysterols, such as 20S, 22S, and 22R, have been tested and showed that these oxysterols are able to regulate the differentiation of MSC such as the M2-10B4 pluripotent marrow stromal cell line (M2 cells), causing a shift from an adipogenic to an osteogenic lineage [23,29,30,31]. Specific oxysterols inhibit adipogenic differentiation of M2 cells by inhibiting the expression of various adipogenic genes such as the adipocyte-specific fatty acid binding protein 2 (*Ap2*) and lipoprotein lipase (*Lpl*) [23]. Moreover, 20S has been found to inhibit adipogenesis and expression of *Pparγ* and *C/ebpα* in M2 cells through hedgehog signaling [29]. Specific oxysterols are novel activators of hedgehog signaling; these oxysterols directly bind to Smoothen without a canonical hedgehog activation by hedgehog proteins [30,31]. Hedgehog signaling, an important signaling pathway for embryonic and post-embryonic development, has been recognized as anti-adipogenic and pro-osteogenic signaling in certain cell types. However, there is no study have been performed to investigate anti-adipogenic effects of different oxysterols in C3H10T1/2 cells. 

In this study, we hypothesized that specific oxysterols are able to inhibit adipocyte differentiation and expression of adipogenic transcripts in C3H10T1/2 mouse stromal cells. We report that 25-HC inhibits adipogenic differentiation of C3H10T1/2 cells and is therefore a potential strategy of inhibiting excess fat accumulation and improving skeletal health in bone microenvironment.

## 2. Results

### 2.1. Effects of Different Oxysterols on Lipid Accumulation in C3H10T1/2 Cells

**Figure 1:**
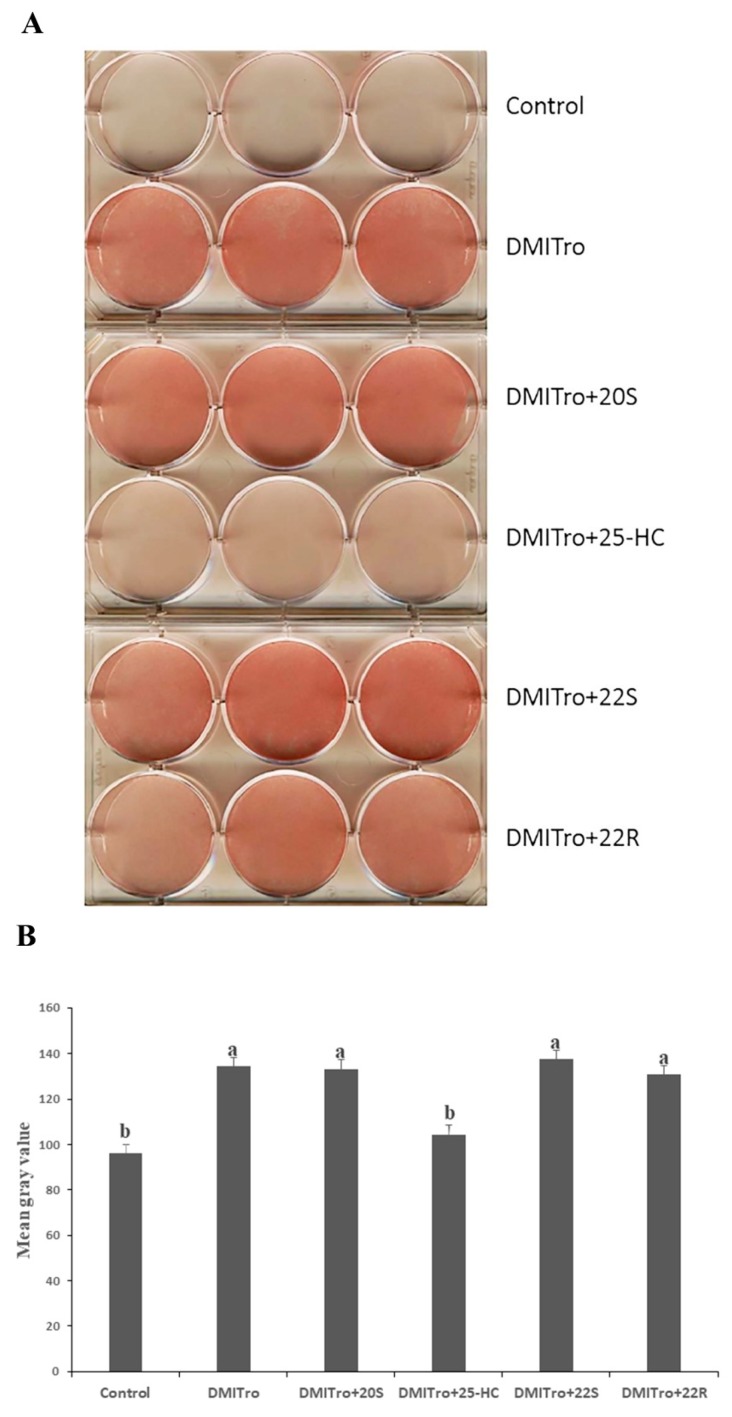
Representative image of C3H10T1/2 mouse cells treated with a control, DMITro (500 nM DEX, 0.5 mM IBMX, 20 µg/mL Insulin and 10 µM Troglitazone), DMITro + 10 µM 25-HC, 20S, 22S or 22R hydroxycholesterols for six days, followed by oil red O staining and scanning of plates for lipid accumulation comparisons. (**A**) oil red O stanning results (**B**) The mean gray value (in pixel) of oil red O staining. The results show the average values of three replicates (*n* = 3) and the SE of the means. Bars with the different letters are significantly different (*p* < 0.001).

To study the effects of specific oxysterols on lipid accumulation, C3H10T1/2 cells were stained with oil red O stain. Treatment of cells with DMITro for six days resulted in a significant accumulation of lipid droplets compared to the control, as shown by the oil red O stained pictures and mean gray value (Figure 1, *p* < 0.001). The lipid droplets were observed from day 2 post treatment and increased with treatment duration. Treatment of cells with DMI or Troglitazone alone did not induce formation of fat droplets in C3H10T1/2 cells. Treatment of cells with DMITro+10µM 25-HC significantly inhibited the adipocyte formation caused by DMITro (Figure 1A,B. *p* < 0.001). Whereas 22R, 20S and 22S did not inhibit formation of lipid droplets compared to DMITro treatment as shown by the oil red O pictures and mean gray value (Figure 1A,B).

### 2.2. Effect of Oxysterols on Expression of Key Adipogenic Genes

**Figure 2:**
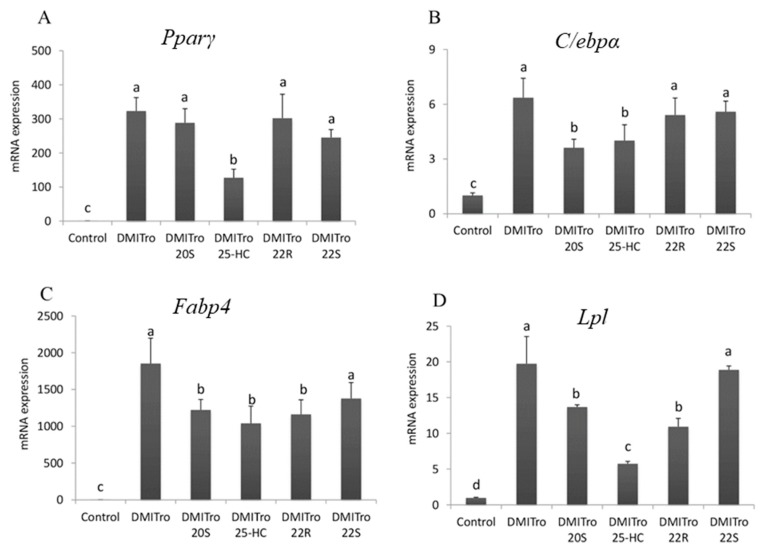
The effects of 25-HC, 20S, 22S and 22R on *Pparγ* (**A**), *C/ebpα* (**B**), *Fabp4* (**C**) and *Lpl* (**D**) mRNA expression induced by DMITro. Cells were treated with a control, DMITro (500 nM DEX, 0.5 mM IBMX, 20 µg/mL Insulin and 10 µM Troglitazone) and DMITro + 10 µM 20S, 25-HC, 22R or 22S for 6 days. The results show the average values of three replicates (*n* = 3) and the SD of the means. Bars with the same letter are not significantly different.

To study the effects of specific oxysterols on the expression of key adipogenic transcripts, quantitative real-time reverse transcription polymerase chain reaction (qRT-PCR) was carried out. Treatment of cells with the DMITro for six days resulted in a significant increase in the mRNA expression of *Pparγ* compared to the control (Figure 2). 25-HC significantly inhibited this increase in *Pparγ* expression induced by DMITro (Figure 2A). However, 20S, 22R and 22S did not significantly inhibit the expression of *Pparγ* after six days of treatment (Figure 2A). The expression of *C/ebpα* was significantly increased by DMITro, and this increase was significantly inhibited by 25-HC and 20S (Figure 2B), while the expression of fatty acid binding protein 4 (*Fabp4)* was significantly inhibited by 25-HC, 20S and 22R hydroxycholesterols (Figure 2C). The expression of *Lpl* was significantly increased by DMITro, and this increase was inhibited by 25-HC, 20S and 22R (Figure 2D) after 6 days of treatment. Of all the oxysterols tested, 25-HC was the most potent in inhibiting adipogenesis and the expression of key adipogenic transcripts in C3H10T1/2 mouse stromal cells as shown in the oil red O staining images and gene expression analysis. 

### 2.3. Effect of 25 Hydroxycholesterol at Different Time Points

**Figure 3:**
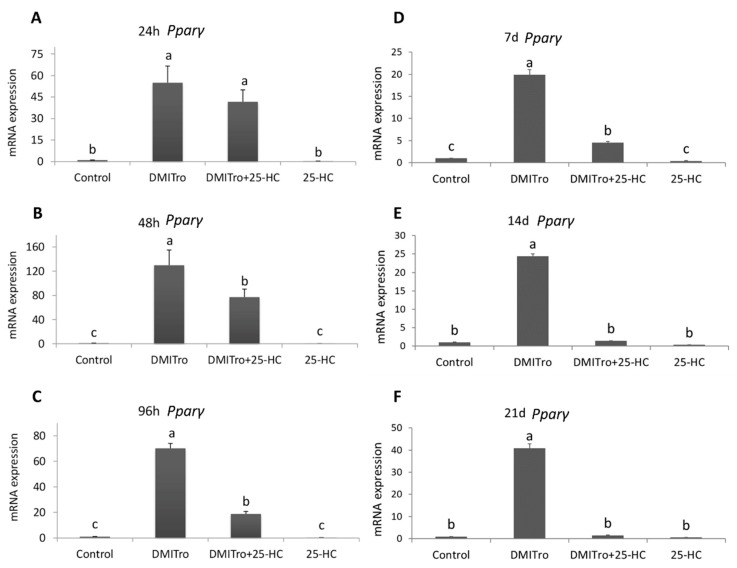
The effects of 25-HC on the expression of *Pparγ* mRNA at different time points: (**A**) 24 hours, (**B**) 48 hours, (**C**) 96 hours, (**D**) 7 days, (**E**) 14 days, (**F**) 21 days. C3H10T1/2 cells at confluence were treated with a control, DMITro (500 nM DEX, 0.5 mM IBMX, 20 µg/mL Insulin and 10 µM Tro), DMITro + 10 µM 25-HC or 25-HC alone for 24 h, 48 h, 96 h, 7 days (d), 14 days and 21 days. The results show the average values of three replicates (*n* = 3) and the SD of the means. Bars with the same letter are not significantly different.

**Figure 4:**
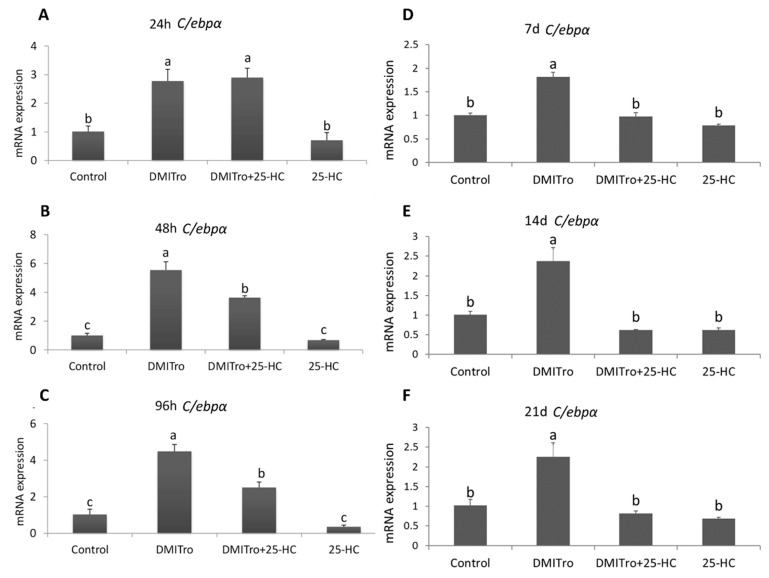
The effects of 25-HC on the expression of *C/ebpα* mRNA at different time points: (**A**) 24 hours, (**B**) 48 hours, (**C**) 96 hours, (**D**) 7 days, (**E**) 14 days, (**F**) 21 days. C3H10T1/2 cells at confluence were treated with a control, DMITro (500 nM DEX, 0.5 mM IBMX, 20 µg/mL Insulin and 10 µM Tro), DMITro + 10 µM 25-HC or 25-HC alone for 24 h, 48 h, 96 h, 7 days (d), 14 days and 21 days. The results show the average values of three replicates (*n* = 3) and the SD of the means. Bars with the same letter are not significantly different.

**Figure 5:**
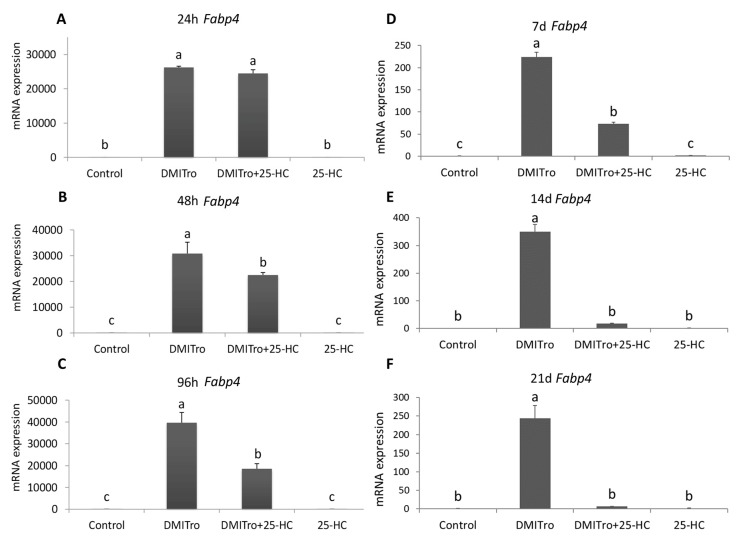
The effects of 25-HC on the expression of *Fabp4* mRNA at different time points: (**A**) 24 hours, (**B**) 48 hours, (**C**) 96 hours, (**D**) 7 days, (**E**) 14 days, (**F**) 21 days. C3H10T1/2 cells at confluence were treated with a control, DMITro (500 nM DEX, 0.5 mM IBMX, 20 µg/mL Insulin and 10 µM Tro), DMITro + 10 µM 25-HC or 25-HC alone for 24 h, 48 h, 96 h, 7 days (d), 14 days and 21 days. The results show the average values of three replicates (*n* = 3) and the SD of the means. Bars with the same letter are not significantly different.

**Figure 6:**
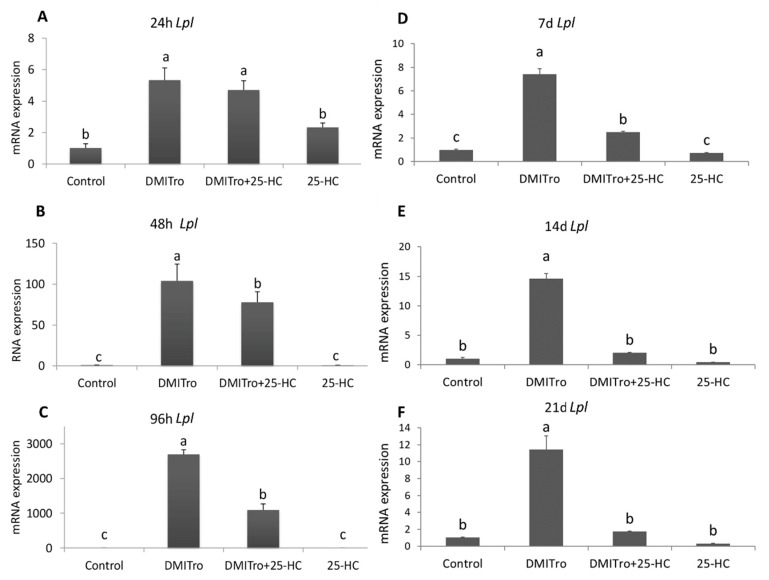
The effects of 25-HC on the expression of *Lpl* mRNA at different time points: (**A**) 24 hours, (**B**) 48 hours, (**C**) 96 hours, (**D**) 7 days, (**E**) 14 days, (**F**) 21 days. C3H10T1/2 cells at confluence were treated with a control, DMITro (500 nM DEX, 0.5 mM IBMX, 20 µg/mL Insulin and 10 µM Tro), DMITro + 10 µM 25-HC or 25-HC alone for 24 h, 48 h, 96 h, 7 days (d), 14 days and 21 days. The results show the average values of three replicates (*n* = 3) and the SD of the means. Bars with the same letter are not significantly different.

To study the effects of 25-HC on the expression of key adipogenic genes at different time points, C3H10T1/2 cells were treated with DMITro in the presence or absence of 25-HC followed by collection of mRNA at 24 h, 48 h, 96 h, 7 days, 14 days and 21 days post treatment. Expression of mRNA was measured by qRT-PCR. Treatment of cells with DMITro caused a significant increase in the expression of *Pparγ* at all time points. This increase in *Pparγ* expression was not significantly inhibited by 25-HC at 24 h (Figure 3A) but was significantly inhibited at 48 h, 96 h, 7 days, 14 days and 21 days (Figure 3B–F). Treatment of cells with DMITro caused a significant increase in the expression of *C/ebpα* at all time points. This increase in expression was not inhibited by 25-HC at 24 h (Figure 4A) but was significantly inhibited at 48 h, 96 h, 7 days, 14 days and 21 days (Figure 4B–F). The expression of *Fabp4* at 24 h was not significantly inhibited by 25-HC (Figure 5A) but was significantly inhibited at 48 h, 96 h, 7 days, 14 days and 21 days (Figure 5B–F). Similarly, 25-HC did not inhibit the expression of *Lpl* at 24 h (Figure 6A) but significantly inhibited it at 48 h, 96 h, 7 days, 14 days and 21 days (Figure 6B–F).

### 2.4. Role of Hedgehog Signalling on the Anti-Adipogenic Effects of 25-Hydroxycholesterol

**Figure 7:**
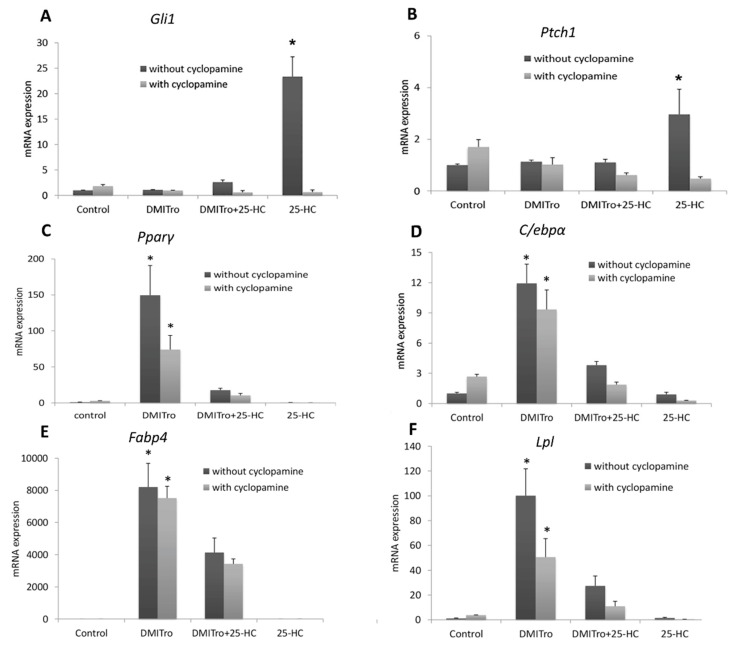
mRNA expression of hedgehog signalling genes: *Gli1* (**A**) and *Ptch1* (**B**); adipogenic genes: *Pparγ* (**C**), *C/ebpα* (**D**), *Fabp4* (**E**) and *Lpl* (**F**), in C3H10T1/2 cells treated with a control, DMITro, DMITro + 25-HC or 25-HC, with or without cyclopamine for 96 h. The results show the average value of three replicates (*n* = 3) and the SD of the means. Bars with * in figure (**A**,**B**) show significant difference between 25-OH treatments with/without cyclopamine (*p* < 0.05); Bars with * in figure (**C**–**F**), show significant difference between DMITro and DMITro + 25-OH regardless of cyclopamine treatments (*p* < 0.05).

**Figure 8:**
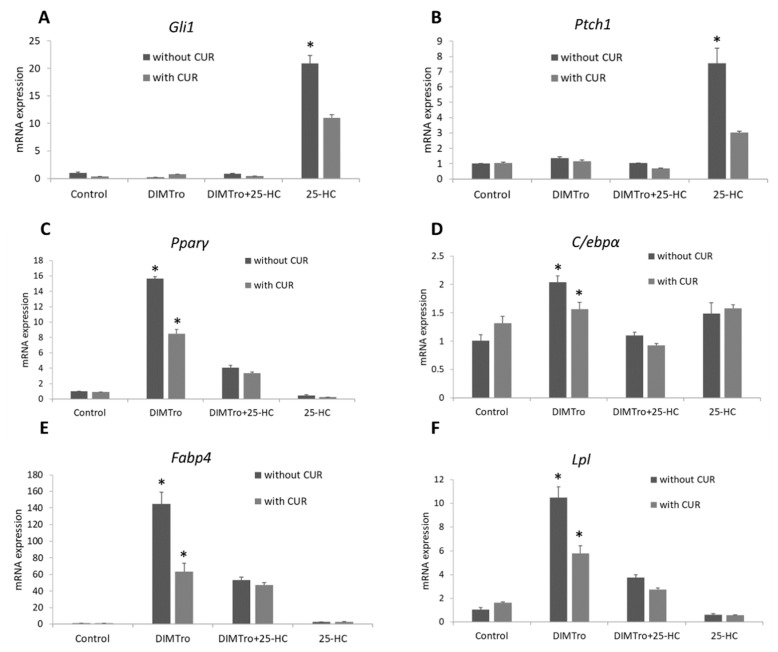
mRNA expression of hedgehog signalling genes: *Gli1* (**A**) and *Ptch1* (**B**); adipogenic genes: *Pparγ* (**C**), *C/ebpα* (**D**), *Fabp4* (**E**) and *Lpl* (**F**), in C3H10T1/2 cells treated with a control, DMITro, DMITro + 25-HC or 25-HC, with or without CUR61414 for 96 h. The results show the average value of three replicates (*n* = 3) and the SD of the means. Bars with * in figure (**A**,**B**) show significant difference between 25-OH treatments with/without CUR61414 (*p* < 0.05); Bars with * in figure (**C**–**F**), show significant difference between DMITro and DMITro + 25-OH regardless of CUR61414 treatments (*p* < 0.05).

To study the mechanism by which 25-HC inhibits adipogenic differentiation in C3H10T1/2 cells, we evaluated the effects of the hedgehog pathway inhibitors, cyclopamine and CUR61414, on 25-HC regulated adipogenesis. C3H10T1/2 cells at confluence were treated with DMITro and DMITro+25-HC, with or without cyclopamine or CUR61414 for 96 h. The effects of cyclopamine and CUR61414 on gene expression were analyzed by qRT-PCR. Treatment of cells with 25-HC alone showed an induction in the expression of *Gli1* and *Ptch1*, which are important mediators of hedgehog signaling. Addition of cyclopamine resulted in inhibition of expression of these two genes (Figure 7A,B and Figure 8A,B). Treatment of cells with the adipogenic media DMITro greatly increased *Pparγ* mRNA expression after 96 h of treatment. Treatment of cells with DMITro + 25-HC significantly inhibited the expression of *Pparγ* compared to the DMITro treatment, while addition of cyclopamine or CUR61414 did not block inhibitory effects of 25-HC on *Pparγ* (Figure 7C and Figure 8C). Similarly, addition of cyclopamine or CUR61414 did not change the expression of *C/ebpα* in cells treated with DMITro+25-HC (Figure 7D and Figure 8D). The same results were observed with the other adipogenic genes *Fabp4* and *Lpl* (Figure 7E,F and Figure 8E,F). 

### 2.5. Role of Liver X Receptor on the Anti-Adipogenic Effects of 25-Hydroxycholesterol

**Figure 9:**
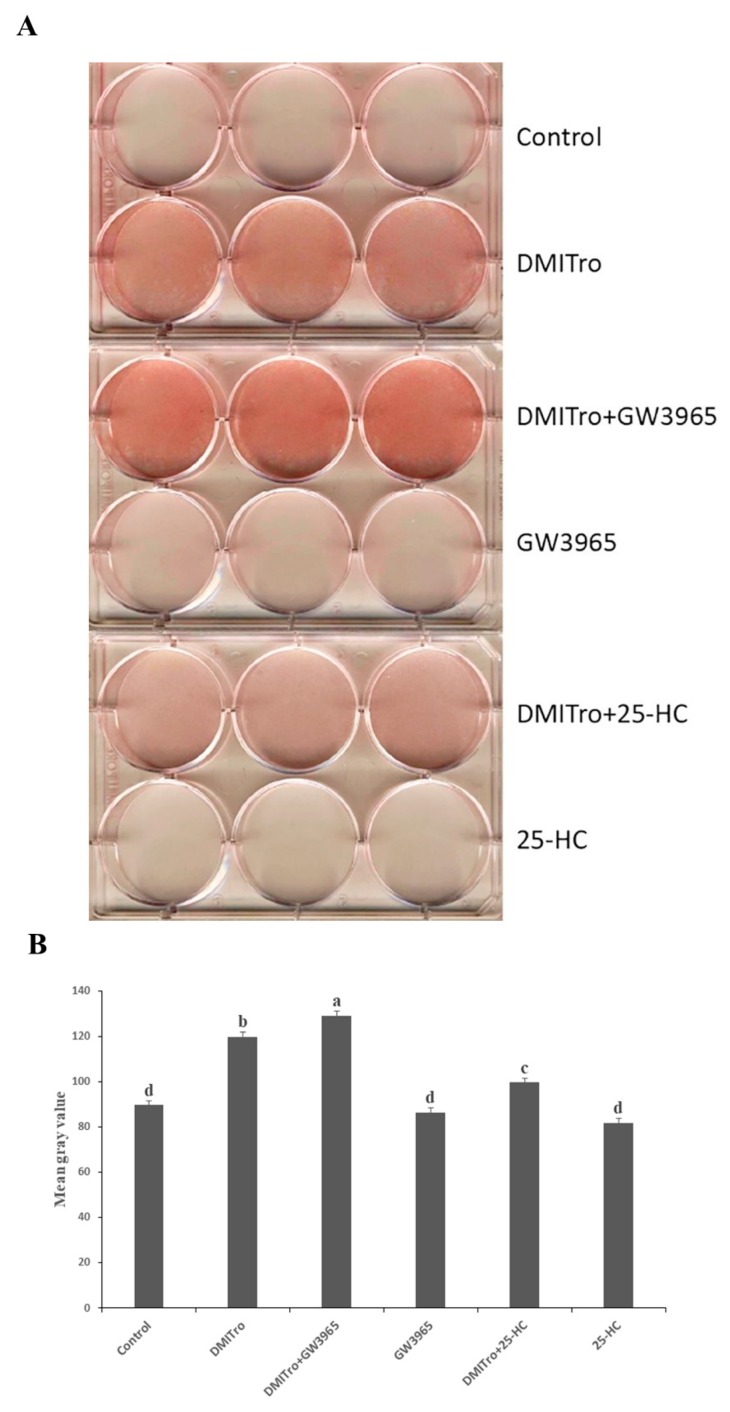
Liver X receptor agonist, GW3965, enhances lipid accumulation in cells treated with the adipogenic media, DMITro. C3H10T1/2 cells at confluence were treated with a control, 2 µM GW3965 or 10 µM 25-HC, with or without DMITro for 96 h, followed by Oil red staining to assess the extent of lipid accumulation in the treatments. (**A**) oil red O stanning results (**B**) The mean gray value (in pixel) of oil red O staining. The results show the average values of three replicates (*n* = 3) and the SE of the means. Bars with the different letters are significantly different (*p* < 0.001).

Liver X receptors (LXRs) are nuclear hormone receptors that play important roles in the regulation of cholesterol and fatty acid metabolism, and are activated by oxysterols including 22R and 20S [32,33]. To assess the possible role of LXR in mediating the anti-adipogenic effects of 25-HC, we examined whether activation of LXR by GW3965, a specific LXR agonist, has effects similar to those of 25-HC in C3H10T1/2 cells. C3H10T1/2 cells at confluence were treated with GW3965 or 25-HC, alone or in combination with DMITro. Oil red O images showed an increase in lipid accumulation in cells treated with DMITro compared to non-treated cells (Figure 9A,B, *p* < 0.001). Treatment of cells with DMITro + GW3965 showed a further increase in lipid accumulation compared to cells treated with DMITro (Figure 9A,B, *p* < 0.001).

**Figure 10:**
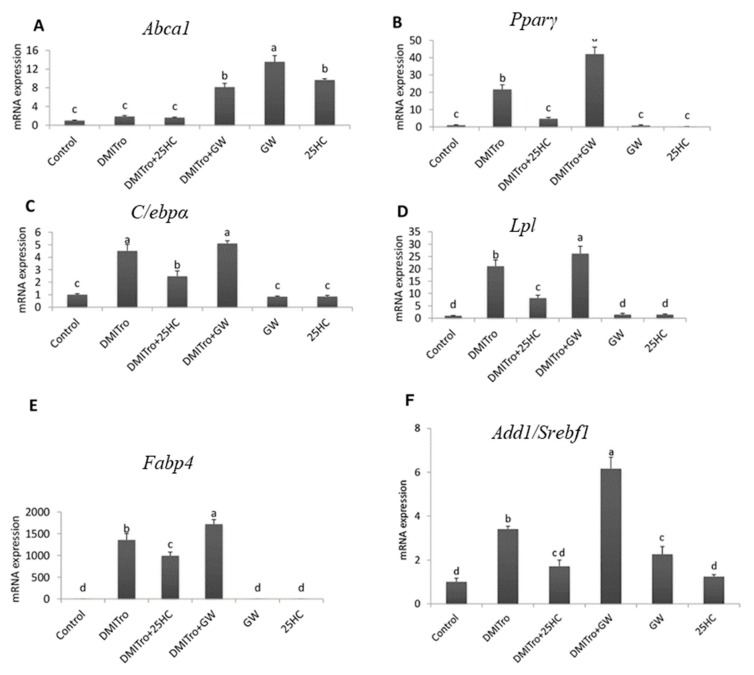
The effect of liver X receptor agonist, GW3965, on the expression of *Abca1* (**A**), *Pparγ* (**B**), *C/ebpα* (**C**), *Lpl* (**D**), *Fabp4* (**E**), and *Add1/Srebf1* (**F**)**.** C3H10T1/2 cells at confluence were treated control, 2 µM GW3965 or 10 µM 25-HC, with or without DMITro, for 96 h. The results show the average values of three replicates (*n* = 3) and the SD of the means. Bars with the same letter are not significantly different.

To assess activation of LXRs in C3H10T1/2 cells, we analyzed the expression of *Abca1*, a target gene of LXR activation [34]. *Abca1* expression was induced in cells treated with GW3965 alone, as well as those treated with DMITro + GW3965 (Figure 10A). *Abca1* expression was also significantly induced in cells treated with 25-HC alone, but not in those treated with DMITro + 25-HC (Figure 10A). Consistent with earlier results, treatment of cells with DMITro caused a significant induction in the expression of *Pparγ*, and addition of 25-HC significantly inhibited this induction. However, in contrast to the effects of DMITro + 25-HC, treatment of cells with DMITro + GW3965 further increased the expression of *Pparγ* (Figure 10B). A similar effect was seen in the expression of *C/ebpα*, *Ap2* and *Lpl* (Figure 10C–E). Thus, the anti-adipogenic effects of 25-HC are not mediated by LXRs since activation of LXRs by GW3965 did not inhibit adipogenesis but instead enhanced the expression of adipogenic genes in C3H10T1/2 cells.

Interestingly, treatment of cells with DMITro induced the expression of Sterol regulatory element binding factor 1/adipocyte differentiation and determination factor 1 (*Add1/Srebf1*; Figure 10F). This induction was significantly inhibited by 25-HC. The *Add1/Srebf1* is a member of the basic helix-loop-helix-leucine zipper (bhlh-lz) family of transcription factors that is associated with adipocyte development and cholesterol homeostasis [35]. The *Srebf* family of transcription factors has been implicated in controlling the expression of *Pparγ* during lipid metabolism [36]. Given that *Add1/Srebf1* regulates adipogenesis through the induction of *Pparγ* expression, it is possible that inhibition of *Add1/Srebf1* expression by 25-HC may lead to inhibition of expression of *Pparγ* and the downstream adipogenic genes.

## 3. Discussion

Mesenchymal stromal cells (MSCs) are able to commit to either adipose, bone, cartilage or muscle lineages upon appropriate induction [21]. MSCs undergo mitotic clonal expansion (MCE) leading to differentiation of preadipocytes to adipocytes [37]. Commitment of MSCs to different cell lineages is as a result of expression of proteins that promote this lineage specific development [38]. Our findings show that the adipogenic cocktail DMITro induces the differentiation of C3H10T1/2 mouse embryonic cells into adipocytes as shown by the accumulation of lipid droplets and expression of adipogenic genes. 

We also demonstrated that 25-HC inhibits adipogenic differentiation of C3H10T1/2 mouse stromal cells. Of the four oxysterols tested, 25-HC proved to be the most potent in inhibiting accumulation of cyctoplasmic lipid droplets and expression of adipogenic protein markers in the cells. 25-HC inhibited the expression of *Pparγ*, which is the main regulator of adipogenesis. In contrast, 20S, 22R and 22S hydroxycholesterols did not inhibit the expression of *Pparγ* after 6 days of treatment. 25-HC also inhibited the expression of *C/ebpα*, an important adipogenic transcription factor, which interacts with *Pparγ* to stimulate the adipogenic differentiation process and is able to activate the promoter region of other genes involved in adipogenesis [6,39]. Fatty acid binding proteins (*Fabps*) including *Fabp4* are important in transport of fatty acids during the early stages of adipocyte differentiation [40]. In this study, treatment of cells with 25-HC significantly inhibited the expression of *Fabp4* after 48 h of treatment. The expression of an adipogenic gene, *Lpl*, was also inhibited by 25-HC after 2 days of treatment. 20S has previously been shown to inhibit *Pparγ* expression in mouse bone marrow stromal cells [29]. The effects of 25HC on apoptosis of MSCs were not mentioned in the current study. However, the viability of cells was not affected by any of treatments. The dosage of oxysterols in the present study was also within the normal dosage compare to previous published studies [41,42]. However, it is worthy to test an apoptosis possibility as an inhibition mechanism of 25-HC in a future study

In the present study, 20S did not inhibit *Pparγ* expression and adipocyte formation in C3H10T1/2 mouse cells. However, 20S was able to inhibit the expression of *C/ebpα*, *Fabp4* and *Lpl*, while 22S did not inhibit the expression of any of the analyzed adipogenic genes. 

We demonstrated that the anti-adipogenic effects of 25-HC on C3H10T1/2 cells are not mediated by Hedgehog (Hh) signalling. Hh signaling controls a number of biological processes including adipogenic differentiation of MSCs [43]. Studies on Hh signalling and adipocyte development are still controversial as different results have been observed depending on cell lines used. Cyclopamine, and CUR61414, specific inhibitors of Hh signalling [43,44,45], are useful in studying the role of Hh in normal development. In the present study we demonstrated that inhibition of the canonical Hh pathway by cyclopamine or CUR61414 does not reverse the anti-adipogenic effects of 25-HC in C3H10T1/2 cells. Hh signalling has been shown to inhibit adipogenesis in 3T3-L1 cells, while inhibition of this pathway increases adipogenesis in the same cells [46]. Down-regulation of Hh pathway has been observed during differentiation of 3T3-L1 cells into adipocytes [47]. A study using 3T3-L1 cells demonstrated that blocking Hh signalling using cyclopamine increases adipogenesis and expression of the adipogenic genes, such as *Pparγ* and *Fabp4* [46].

In contrast, our findings show that blocking Hh signalling with cyclopamine or CUR61414 does not increase adipogenesis or expression of adipogenic genes in C3H10T1/2 cells. These findings are consistent with a study carried out using murine 3T3-L1 cells where inhibition of Hh signalling using cyclopamine did not induce adipogenesis or expression of adipogenic differentiation markers [47]. In our findings, treatment of C3H10T1/2 cells with DMITro increased the expression of *Pparγ*, and addition of cyclopamine or CUR61414 decreased the expression of this marker, suggesting that inhibition of Hh signalling does not trigger adipogenesis in these cells (Figure 7C and Figure 8C). Treatment of C3H10T1/2 cells with DMITro + 25-HC decreased the expression of *Pparγ* compared to the DMITro treatment. Addition of cyclopamine or CUR61414 did not block inhibitory effect so 25-OH on *Pparγ* expression, suggesting that the anti-adipogenic effects of 25-HC are not mediated through the canonical Hh signalling (Figure 7C and Figure 8C). Similar results were observed with *C/ebpα*, *Fabp4* and *Lpl* (Figure 7D–F and Figure 8D–F). Although we did not investigate non-canonical pathways in the current study, non-canonical pathways may involve in anti-adipogenic effects of oxysterols. In 3T2-L1 cells, hedgehog signaling reduced adipogenic differentiation via AMP-activated protein kinase regulated by a non-canonical pathway [48]. In the future, it is worthy to evaluate anti-adipogenic effect of oxysterols through non-canonical pathways. 

We also showed that the anti-adipogenic effects of 25-HC are not mediated by LXRs. LXRs are important in the regulation of cholesterol, where they regulate a set of genes associated with cholesterol catabolism, absorption and transport [32,33,49]. In addition, LXRs also regulate several genes involved in fatty acid metabolism by either regulating the expression of *Add1/Srebf1* or by directly binding the promoters of specific lipogenic genes [50,51,52]. Naturally-produced oxysterols such as 22R and 24S hydroxycholesterol have been shown to activate LXRs [53,54]. In the present study, there was an increase in adipogenesis in cells treated with the LXR agonist, GW3965 in combination with DMITro as seen in the oil red O images (Figure 9). Gene expression results showed that both 25-HC and the LXR agonist GW3965 activated the LXRs in undifferentiated C3H10T1/2 cells as shown by the induction of expression of *Abca1* (ATP-binding cassette sub-family A, member 1) gene, an important marker of LXR activation (Figure 9A). However, in the presence of the adipogenic cocktail DMITro, 25-HC did not induce the expression of *Abca1* gene, suggesting that there may be an additional interaction mechanism between DMITro-induced adipogenesis and anti-adipogenic action of 25-HC. In contrast, GW3965 was still able to induce the expression of *Abca1* in the presence of DMITro. In the analysis of adipogenic genes, activation of LXRs by GW3965 did not have effects similar to those of 25-HC. In contrast to 25-HC, LXR activation by GW3965 led to an increase in the expression of adipogenic genes, indicating that the anti-adipogenic effects of 25-HC are not mediated by LXRs. These findings are similar to a study performed using M2-10B4 cells where the osteogenic effects of 20S and 22R on M2 cells were found to be independent of the LXR activation since activation of LXRβ by the pharmacological agent TO-901317 did not yield effects similar to those of 20S and 22R [23].

In the present study, we found that the anti-adipogenic effects of 25-HC on C3H10T1/2 cells could be mediated in part through inhibition of *Add1/Srebf1*. Expression of *Add1/Srebf1* has been shown to augment adipogenic differentiation through direct induction of *Pparγ* gene expression as well as through production of endogenous *Pparγ* ligands [36,55]. Ectopic expression of *Add1/Srebf1* in 3T3-L1 and HepG2 cells induces endogenous *Pparγ* mRNA levels [36]. Furthermore, ectopic expression of a dominant-negative *Add1* in 3T3-L1 cell line was observed to inhibit adipocyte differentiation and expression of adipocyte-specific genes, while expression of the active form of *Add1* exhibited more lipid accumulation in the cells [33]. In the present study, treatment of C3H10T1/2 cells with the adipogenic cocktail DMITro induced the expression of *Add1/Srebf1*, and addition of 25-HC significantly inhibited the expression of *Add1/Srebf1*. Since 25-HC inhibited the expression of *Add1/Srebf1* and given that expression of *Add1/Srebf1* is important in augmenting adipogenic differentiation and expression of *Pparγ* and the downstream adipogenic genes, it is possible that the anti-adipogenic effects of 25-HC on C3H10T1/2 cells may be mediated through inhibition of *Add1/Srebf1*. Thus, it is necessary to elucidate *Add1/Srebf1*-mediated inhibitory mechanisms of 25-HC on adipogenesis and *Pparγ* in C3H10T1/2 cells in a future study. 

Together, our results show that 25-HC inhibits adipogenic differentiation in C3H10T1/2 cells by inhibiting accumulation of cyctoplasmic lipid droplets and expression of core adipocyte markers, including *Pparγ*, *C/ebpα, Fabp4*, and *Add1/Srebf1*. 25-HC may thus be useful in providing an intervention in excess fat cell formation associated with obesity and osteoporosis. We also report that the anti-adipogenic effects of 25-HC in C3H10T1/2 cells are not mediated through hedgehog signalling since inhibition of this pathway by cyclopamine or CUR61414 does not reverse the anti-adipogenic effects of 25-HC. However, it is possible that the inhibitory effects of 25-HC on adipogenic differentiation may be mediated through the *Add1/Srebf1* pathway since 25-HC inhibits the expression of *Add1/Srebf1*, a transcription factor that plays a role in the activation of *Pparγ* mRNA expression.

## 4. Materials and Methods 

### 4.1. Reagents

Oxysterols, oil red O stain and GW3965 were purchased from Sigma-Aldrich (St Louis, MO, USA). Dexamethasone (DEX), Insulin and 3-Isobutyl-1-methylxanthine (IBMX), cyclopamine and purmorphamine were purchased from Cayman chemical company (Ann Arbor, MI, USA). Troglitazone was purchased from Tocris Bioscience (Ellisville, MO, USA). Dulbecco’s modified Eagle’s medium (DMEM), Fetal bovine serum (FBS), Penicilin, streptomycin and L-glutamate were purchased from Mediatech, Inc (Manassas, VA, USA).

### 4.2. Cell culture

C3H10T1/2 mouse embryonic stromal cells were purchased from ATCC (Rockville, MD, USA) and cultured in DMEM supplemented with 10% FBS, 100 U/mL penicillin, 100 μg/mL streptomycin and L-glutamate, and incubated at 37 °C in a 5% CO_2_ incubator until confluency. The cells were then induced to differentiate into adipocytes in the presence of an adipogenic cocktail (DMITro), consisting of 500 nM DEX, 0.5 mM IBMX, 20 µg/mL Insulin and 10 µM Troglitazone (Tro). Inhibition of adipogenesis was induced by use of oxysterols as follows; DMITro + 10 µM 20S, 25-HC, 22R or 22S hydroxycholesterol with 3 replications per treatment (*n* = 3). The control treatment consisted of 10% FBS in DMEM. The cells were re-treated after 2 days (without DEX and IBMX) and thereafter re-treated after 3 days. RNA was collected after 6 days of adipogenic differentiation.

### 4.3. Oil Red Staining

To examine lipid accumulation and formation of fat droplets, the cells were fixed with 60% isopropanol and stained with oil red O stain for 20 min. The stain was then rinsed off, and the plates were allowed to air-dry [35]. Staining results pictures were taken at same exposure settings. The pictures were then converted to 8-bit gray scale using Image J software (1.48v version, U. S. National Institutes of Health, Bethesda, MD, USA). The mean gray value (300 mm^2^ area) was measured inside of each well. The values were presented as a number between 0 (black)-255 (white). The lower value represents darker staining.

### 4.4. RNA Extraction and Quantitative Real Time PCR (qRT-PCR)

Total RNA was extracted using TRIzol (Invitrogen, Burlington, ON, USA) according to the manufacturer’s instruction. RNA (2 µg) was reverse-transcribed to cDNA by reverse transcription polymerase chain reaction (RT-PCR) analysis using high capacity cDNA synthesis kits according to the supplier’s protocol (Applied Biosystems, Burlington, ON, USA). qRT-PCR was performed on CFX Connect TM Real-Time PCR Detection instrument (Biorad, Hercules, CA, USA). All real-time PCR samples were prepared in duplicates and analyzed by real-time PCR using iTaq^TM^ Universal SYBR Green Supermix (Bio-Rad, Hercules, CA, USA). Primers (Table 1) and cDNA templates were subjected to qRT-PCR at 95 °C for 10 min, followed by 40 cycles of 15 s denaturation at 95 °C, 20 s annealing, and 15 s extension at 72 °C, followed by 95 °C for 15 s and a melt curve The gene expression data were generated using the ∆∆Ct method where expression of target genes were normalized to the expression of the house keeping gene, glyceraldehydes-3-phosphate dehydrogenase (GAPDH). 

### 4.5. Statistical Analysis

The generated data were analyzed using the General Linear Model (GLM) procedure of the Statistics Analysis System (SAS) Institute version 9.2. Differences between the groups were compared by one-way ANOVA and subsequent Tukey’s studentized range test. A probability value of < 0.05 was considered significant.

## Figures and Tables

**Table 1:** Primer sequences for PCR reactions.

Gene	Primer Sequences (Forward and Reverse)	Accession Number
*Gapdh*	5′-ATGGACTGTGGTCATGAGCC-3′	NM_001289726.1
	5′-ATTGTCAGCAATGCATCCTG-3′	
*Pparγ*	5′-TGAAACTCTGGGAGATTCTCCTG-3′	NM_011146.3
	5′-CCATGGTAATTTCTTGTGAAGTGC-3′	
*C/ebpα*	5′-GGACAAGAACAGCAACGAGTACC-3′	NM_001287514.1
	5′-GGCGGTCATTGTCACTGGTC-3′	
*Fabp4*	5′-AACACCGAGATTTCCTT-3′	NM_024406.2
	5′-ACACATTCCACCACCAG-3′	
*Lpl*	5′-AGGACCCCTGAAGACAC-3′	NM_008509.2
	5′-GGCACCCAACTCTCATA-3′	
*Add1/Srebf-1*	5′-CCTCCACTCACCAGGGTCT-3′	NM_011480.3
	5′-CTCAGCAGCCCCTAGAACAA-3′	
*Abca1*	5′-CTGTGTTGTGTGGGCTCCTC-3′	NM_013454.3
	5′-GTCAGCGTGTCACTTTCATGG-3′	
*Gli1*	5′-GCTTGGATGAAGGACCTTGTG-3′	NM_010296.2
	5′-GCTGATCCAGCCTAAGGTTCTC-3′	
*Ptch1*	5′-TTCTGCTGCCTGTCCTCTTATC-3′	NM_008957.2
	5′-CCTGCTGTGCTTCGTATTGC-3′	

## Abbreviations

*Abca1*	ATP-binding cassette sub-family A, member 1
*Add1/Srebf1*	Adipocyte differentiation and determination factor 1/sterol regulatory
*C/ebpα*	CCAAT/enhancer binding protein α
*Fabp4*	Fatty acid binding protein 4
*Gapdh*	Glyceraldehyde-3-phosphate dehydrogenase
*Lpl*	Lipoprotein lipase
LXR	Liver X Receptor
MSC	Mesenchymal stromal cells
*Pparγ*	Peroxisome proliferator-activated receptor γ
T2DM	Type 2 diabetes mellitus
